# A Focus Group on Dental Pain Complaints with General Medical Practitioners: Developing a Treatment Algorithm

**DOI:** 10.1155/2016/4760672

**Published:** 2016-07-04

**Authors:** Ava Elizabeth Carter, Geoff Carter, Robyn Abbey

**Affiliations:** ^1^Griffith University, Parklands Drive, Southport, QLD 4215, Australia; ^2^Department of Employment and Human Relations, Parklands Drive, Southport, QLD 4215, Australia

## Abstract

*Objective*. The differential diagnosis of pain in the mouth can be challenging for general medical practitioners (GMPs) as many different dental problems can present with similar signs and symptoms. This study aimed to create a treatment algorithm for GMPs to effectively and appropriately refer the patients and prescribe antibiotics.* Design*. The study design is comprised of qualitative focus group discussions.* Setting and Subjects*. Groups of GMPs within the Gold Coast and Brisbane urban and city regions.* Outcome Measures*. Content thematically analysed and treatment algorithm developed.* Results*. There were 5 focus groups with 8-9 participants per group. Addressing whether antibiotics should be given to patients with dental pain was considered very important to GMPs to prevent overtreatment and creating antibiotic resistance. Many practitioners were unsure of what the different forms of dental pains represent. 90% of the practitioners involved agreed that the treatment algorithm was useful to daily practice.* Conclusion*. Common dental complaints and infections are seldom surgical emergencies but can result in prolonged appointments for those GMPs who do not regularly deal with these issues. The treatment algorithm for referral processes and prescriptions was deemed easily downloadable and simple to interpret and detailed but succinct enough for clinical use by GMPs.

## 1. Introduction

A number of studies have identified that medical practitioners have only a small or no amount of training during medical school in regard to treating dental infections or dental pain [1–4]. A review of management of dental emergencies by general medical practitioners (GMPs) published in 2011 identified that many countries lack an education curriculum for their medical students regarding dental emergencies [5]. Even many registered medical practitioners had still not received any form of dental emergency education in the form of Continued Professional Education (CPD) [6], with one study noting that most respondents had only 2 or less hours of preventive dental education [7]. Appropriate and accurate knowledge of dental infections appears to thus be necessary to enable suitable referral [8, 9], whether that is a general dental practitioner, a dental specialist, or the emergency department.

Without a doubt, suitable knowledge of any condition afflicting a patient is necessary to correctly diagnose, refer, and prescribe. Indeed the differential diagnosis of pain in the oral cavity can be very challenging as many different types of dental problems can present with similar signs and symptoms and sometimes the pain may not even originate from the dentition [10]. Family medical practitioners do not have the specific dental instruments that the general dentist has to help diagnose and treat different complaints. Considering that the aim of the GMPs is not to treat but to determine the next phase of referral it was hypothesised that a treatment algorithm would efficiently and effectively enable GMPs to identify the basic nature of the patients' complaint and refer accordingly. Currently there are no published treatment algorithms on this subject for GMPs.

The objectives of this study were to create a simple but accurate treatment algorithm to help practitioners in the process of diagnosing, referring, and prescribing pain relief and/or antibiotics for patients who present complaining of dental pain.

## 2. Methodology

This study utilised an exploratory qualitative design, using a convenience sampling technique with focus group discussions following the 2013 Declaration of Helsinki for medical ethics and was conducted from the period of April 2014 to December 2014. This study aimed to identify what GMPs would prefer in a treatment algorithm to effectively and appropriately refer, tentatively diagnose, and prescribe antibiotics (excluding mouth ulcers and trauma). The stakeholder group was general medical practitioners.

### 2.1. Study Participants

To find participants, 209 Family General Practice clinics were randomly contacted using information retrieved from the Australian Health Practitioners Regulation Agency (AHPRA) via letters sent to each clinic. Information about the study, consent forms, and ethics information were included in the original letters sent to the 209 clinics. Forty-three (*N* = 43) individual practitioners responded (20% response rate). The 43 practicing GMPs that accepted the invitation to participate were mailed a short questionnaire to fill out before attending the focus groups. The questionnaire included information on their age group, average years of experience, whether they see indigenous patients, and whether they use Therapeutic Guidelines daily. All 43 participants sent back the completed questionnaire in the prepaid envelopes.  Table 1 summarises the focus groups participant profile.

### 2.2. Focus Group Sessions

The facilitators conducted the discussions and audiotaped them following written consent; any potentially personal data was deleted. Focus groups were continued until saturation was achieved with 5 focus groups of 8-9 GMPs. Saturation was determined using the theory published by Cameron (2005) [12]. In this study, once focus groups began presenting significant repetition (>80%) of the previous themes noted from prior groups and no new information was shared, then saturation was reached. The facilitator (a registered dental practitioner, a registered General Practitioner, and a psychologist) acted as a stimulant for discussion, not experts, guiding the groups to answer three open-ended questions for reflection, summary, and clarification:

1. What types of dental pain complaints do you commonly see in everyday practice and how do you manage them?
2. Would you change your management of these complaints if you knew the accepted therapeutic guidelines about dental pain?
3. What sort of diagnostic aid would most benefit your everyday practice and how would you design it?

### 2.3. Data Analysis

Audiotapes data was transcribed verbatim independently by two of the authors, crosschecked, and then analysed with thematic content analysis and checked using ATLASti (4.2) to categorise the transcripts [13].

### 2.4. Data Trustworthiness and Reflexive Analysis

Informal member (off-record) checking was done throughout the focus groups to clarify, summarise, and paraphrase. The discussion supporting the data should assist the reader in evaluating the trustworthiness of this study after analysis, member checking was performed. Here authors sent the de-identified data to two previously identified GMPs who were happy to review the data. The authors considered the effect of reflexivity on this qualitative research, in particular that it was qualitative research in dentistry [14, 15]. Although the researchers were part of the process of discussion, there was a point made in the methodology for the facilitator not to provide their own point of view on the questions to avoid direct bias. In focus group 4 there were many questions forwarded to the facilitator as to what she thought of antibiotics resistance in dentistry, as the participants were aware she was a general dental practitioner. The paraphrasing regarding this theme from this group was removed, as honest opinions may not have been expressed for fear of professional judgement.

## 3. Results

Five focus groups were conducted from the period of April to December 2014 (see Table 1) consisting of general medical practitioners. Results were framed by two overarching themes: evidence based practice and preferred algorithm design. Results are presented using these themes.

### 3.1. Evidence Based Practicing

The most common form of dental pain presenting to the general medical practitioners in this study was large swellings or abscesses in the oral cavity. Participants spent time discussing which presentations of pain they commonly saw. Specific concerns of the practitioners included correct referral, appropriate prescription of analgesics and antibiotics, and whether or not the patient would have access to a dentist in time. Almost all practitioners related these concerns back to a lack of continued professional development regarding emergency dental problems. In addition, practitioners felt there was not enough information about who to refer to or which forms of dental pain require antibiotics and if these antibiotics would actually penetrate into the tooth infection.

Uncertainty of whether or not they were providing the appropriate prescriptions was a common theme:

*“We were just never taught this sort of thing at medical school.”*

*“We don't actually get any information from drug companies about what to give for dental infections.”*

*“I always give amoxicillin, just in case, even if I don't know what the problem is.”*

*“Usually the patients say they can't afford a private dentist, I see mostly pensioners, so I give antibiotics and analgesics and hope that the public dental hospital can see them sometime soon.”*

It also appeared that GMPs thought there was a significant call for continued professional education on the subject of dental emergencies in the medical setting.

*“It would be better if we had some form of free information pack from a dental group outlining who we should refer to.”*

One particular practitioner summarised the education issue succinctly:

*“I don't think we need to teach the new medical students dental issues, but I think there does [sic] need to be more CPD courses for us to do so that we have the opportunity to learn about these issues, especially if we're working in areas of low SES and poor dental access.”*

Participants believed that if they had some more knowledge on the different symptoms oral pain can present with, then they would be able to triage which patients were emergencies versus which patients needed a general dental checkup. They also mentioned that they felt dentists were not involved as much as they should be with patients as GMPs are and that more contact between GMPs and dentists would benefit both disciplines and increase knowledge of best evidence based practice. Dentists were seen as a valuable source of information:

*“I think there's always been a divide [sic] between dentists and doctors, I don't know why, but it needs to change”*

*“I think that it would be good if dentists could get together and publish a guideline for doctors so that we know the basics of dental pain so we can do the right thing.”*

*“If we could exchange views on the subject then we would be able to treat patients more effectively. I'm not saying this is easy in private practice, but it's probably do-able in the public system.”*

### 3.2. GMP's Preferred Algorithm Design

When discussing what sort of treatment algorithm design GMPs would prefer, there was at first a little confusion. A few doctors knew exactly what they wanted, but the majority of participants did not know what they would want included and what might be unnecessary.

*“I would like to see a linear progression graph, where we can move down and link up the symptoms to the cause.”*

*“I'm only assuming, but if there are some situations of dental pain that don't need antibiotics then I'd like the algorithm to include whether or not we have to give antibiotics.”*

Two focus groups brought up the fact that they often see indigenous patients and that they usually only come to the GMPs if they have a large swelling of abscess. They focused on whether or not certain types of dental infections require referral to the emergency room. All participants understood the signs and symptoms of airway obstruction but not all participants knew whether or not to refer to the emergency room if there was a large swelling but no airway obstruction.

*“Mostly we see indigenous patients in our practice. They have poor oral health care and low access to dental services and so when they do see us they're in a lot of pain, and often have a large swelling. But I'd like to know which swellings need immediate referral and which ones don't”*

*“I think it would be nice to have a comprehensive graph, one that I can follow down from symptoms to a likely diagnosis then at the end it also includes whether antibiotics are requited and if emergency referral is needed. I think as qualified medical practitioners we know when a medical emergency comes out way, but it's nice to be able to say we have an information source.”*

**Figure 1:**
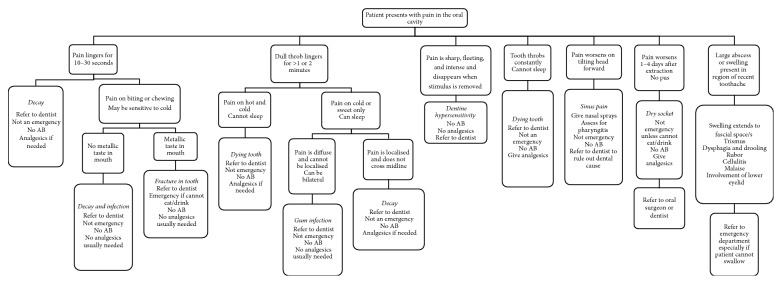
Treatment algorithm for dental infections for medical practitioners [11].

After in-depth discussion amongst participants, all but one focus group provided a draft of possible algorithms to the facilitators. This basic picture of the algorithm was taken by the researchers and provided the basis for the algorithm provided in Figure 1 and Table 2. The researchers analysed the possible conditions to include in the algorithm, which aimed to encapsulate all the useful points as outlined by the participants, showing each common pain presentation, with a linear progression of symptoms that leads to a diagnosis, and identifying the need for antibiotics, analgesics, or emergency referral. The citations given were searched for using PubMed using search terms “dental pain”, “dental antibiotics”, and “dental emergencies in medicine” and the following citations were used to define the algorithm [7–10, 12–14]. Only articles published in English and within an Australasian context were included in the study. The Dental Therapeutic Guideline (2012) was already known to the researcher (previous training) but online copies were cited [11]. A table was also created to act as a supplement with the algorithm, as suggested by a participant, so as to show which antibiotics are appropriate; this table is based on the dental therapeutic guidelines which describe for dental practitioners which antibiotics should be used depending on patients severity of infection [12].

## 4. Discussion

### 4.1. Issues with the Methods

The strengths of this study were its relatively high sample size, and that selection of participants was unbiased, and that 44% of participants reported that at least 50% of their patient load were indigenous patients. Many GMPs (73%) reported using Therapeutic Guidelines (TG) on a daily basis; however, it was not known which TG was used. Limitations included the long period of data collection and the fact that a number of groups of GMPs were not included in the study. For example, it was not specifically known for how long each practitioner had worked, as the pre-focus group questionnaire only asked for an average timeline of work experience (e.g., 1–10 years). Inclusion of an equal number of experienced GMPs and new graduates would have given the focus groups a broader range of discussion points as to how they gathered information using evidence based medicine such as using the internet, articles, or textbooks. Utilising a purposive sampling technique would enable a wide range of GMPs to participate, and possible targeting for gender, ethnicity, religion, political views, age, and education could be controlled for, but further observations would probably only yield minimal information.

The results of the analysis showed that the participants had a wide range of experiences with dentists and patients with dental infections, and the algorithm was designed to reflect this wide range of possible presenting complaints. Results appear to be transferable to different groups of GMPs, ranging from those with little to no experience with dental infections to those that have worked in practices that see dental emergencies daily such as emergency departments. Post-analysis member checking identified some distortions in the data that were removed and validated the views of the focus groups. A number of participants noted that their patient base consisted of equal to or more than 50% of indigenous patients. Considering that the most common dental problem experienced by medical practitioners was large swellings or abscesses, this may indicate the level of dental access in these areas. The algorithm may therefore be applicable to rural and remote areas which statistically have more instances of abscesses, but further validation of the algorithm is recommended [16]. In addition, caution should be used when applying this algorithm to practices with patients that use English as a second language (ESL) as it may be difficult to elicit the exact meaning of the patients' chief complaint.

Continued professional development is mandatory for medical practitioners worldwide and involves practitioners taking online or hands-on courses in certain medical disciplines to maintain up-to-date knowledge in clinical practice. Many GMPs felt that there were not enough development courses available regarding dental infections and dental emergencies. The United States currently has a well-established module to address CPD for dental emergencies and this could be modified for Australian and other national guidelines [17]. A guide to dental infections from a medical practitioner's point of view may help close the gap in this knowledge as has been alluded to by previous researchers [1]. It also appears that GMPs feel that dentists and doctors are not communicating as much as they could be and that further interdisciplinary communication could aid in patient care and treatment.

As a general dental surgeon, the main author may have introduced bias into the results but overall the data was considered to be overall unaffected by the facilitator.

It is obvious that medical practitioners already understand what sort of situations require emergency referrals but it appears that reinforcement of the signs and symptoms of dental emergencies would be useful and this has been designed and provided to work in conjunction with treatment algorithm.

### 4.2. Interpreting the Treatment Algorithm

The purpose of the treatment algorithm was to provide medical practitioners with a greater understanding of the signs and symptoms each dental condition can present with. Many of the GMPs at the focus groups (72%) requested that a text-based interpretation of the algorithm be supplied. Its purpose was not to act as a sole diagnostic tool but to aid in situations when there is confusion as to who to refer to and whether or not the case is a true dental or surgical emergency.

#### 4.2.1. Pain Lingering for 10–30 Seconds after Stimulus Is Removed

Any pain lingering for less than one minute does not generally involve the nerve. Stimulus is cold or sweet but can also include acidic and spicy ones [10]. When stimulus involves biting, then diagnosis is likely to involve fracture in the tooth [18]. When there is also a metallic taste, the likelihood of a true fracture somewhere within the tooth is increased [19]. Referral to a dentist is necessary. Antibiotics are not indicated [11].

#### 4.2.2. Dull Throb Lingers for >1 or 2 Minutes after Stimulus Is Removed

There are a few different causes of this presentation of pain. Pain on hot stimuli and inability to sleep represent severe inflammation of the nerve [20]. Pain which is on cold only and cannot be localised is most likely a gum infection. Pain which is on cold only and can be localised is likely to be a decay. None are surgical emergencies and need referral to a dentist. Antibiotics are not indicated, but NSAIDS are particularly useful if the medical history allows them [11].

#### 4.2.3. Pain Is Sharp, Fleeting, and Intense and Disappears When Stimulus Is Removed

Fleeting, intense, and sharp pain occurring on cold or sweet that almost immediately disappears when stimuli is removed is pathognomonic of dentine hypersensitivity [10]. Referral to a dentist is necessary but it is not a surgical emergency. Antibiotics and analgesics are not indicated [11].

#### 4.2.4. Tooth Throbs Even without Stimulus

Throbbing tooth without initiating stimuli is characteristic of a symptomatic necrotic nerve and requires referral to a dentist but it is not a surgical emergency [20]. Antibiotics are not indicated, but NSAIDS are particularly useful if the medical history allows them [11].

#### 4.2.5. Pain Worsens on Tilting Head Forward

Pain on tilting one's head forward and the absence of any other dental pain are pathognomonic of an inflammation of the maxillary or frontal sinus. Antibiotics, steam inhalation, and nasal sprays/solutions are useful [11]. This is not a surgical emergency. Referral to a dentist is necessary to rule out dental origin.

#### 4.2.6. Pain Worsens 1–4 Days after Extraction

Pain worsening 1–4 days after extraction is most likely due to a dry socket (alveolar osteitis) which is a lack of healing and not a bacterial infection. Antibiotics are not indicated. The socket can be rinsed with saline solution to remove debris. A dressing of eugenol and iodine (Alvogel*™*) is very effective [11]. Pain relief is warranted. Referral back to treating surgeon is necessary, but it is not a surgical emergency.

#### 4.2.7. Large Abscess or Swelling Present in Region of Recent Toothache

Any abscess or swelling requires timely referral. Conditions that compromise the airway or nervous system or where a delay could result in the death or permanent impairment of health are surgical emergencies [21]. Hence, any swelling that has led the patient to present with trismus (difficulty opening mouth) or dysphagia or involves the lower eyelid should be referred to the emergency department and the maxillofacial unit associated. If the infection is causing trismus, drooling of saliva, and dysphagia, then there is likely involvement of one or more of the submandibular, submasseteric, lateral pharyngeal, or pterygomandibular spaces and it is a surgical emergency [22]. If the infection progresses past this point or is allowed to linger, the following serious complications can occur: (i) upper airway obstruction due to anterior displacement of posterior pharyngeal wall and elevation of tongue, (ii) rupture of abscess into retropharyngeal or lateral pharyngeal space causing aspiration of pus and consequent asphyxiation, and (iii) extension inferior to the diaphragm involving the mediastinum [22]. Any involvement affecting the lower eyelid threatens to extend into the infraorbital space potentially causing cavernous sinus thrombosis and/or permanent damage to vision [22]; this also requires immediate referral to the emergency department and maxillofacial unit associated.

## 5. Conclusion

Diagnosis of dental pain can be difficult, especially when one has not had any formal training. The treatment algorithm presented gives general family practitioners a simple method to determine oral pain typically presenting to the medical office and may aid in saving time. The majority of the general family practitioners within the focus group believed the treatment algorithm had clinical and theoretical uses and it benefited them during consultations. All cases require referral to a dentist or the emergency department or maxillofacial unit, to determine the definitive diagnosis, but the physician and the patient may benefit from a more accurate referral and peace of mind regarding the cause. Followup research as to the long-term utility of this treatment algorithm would be useful to determine its validity.

## Figures and Tables

**Table 1:** Profile of participants in focus groups.

	General medical practitioners (*N* = 43)								
Number of focus groups	Participants per group	Female	Male	<10 years in practice	10–20 years in practice	>20 years in practice	Participants average age group	Number of participants using therapeutic guidelines daily	Number of doctors whose workload included 50% indigenous persons
5	8-9	19	24	11	9	23	40–50 years	31	17

**Table 2:** Antibiotics and pain relief for dental pain presenting to the general family practitioners [11].

Condition	Antibiotic cover	Analgesic cover
Severe superficial infections with swelling and/or systemic signs and symptoms	Amoxycillin 500 mg (child: 12.5 mg/kg up to 500 mg) orally, every 8 hours for 5 days *OR* Phenoxymethylpenicillin 500 mg (child: 12.5 mg/kg up to 500 mg) orally, every 6 hours for 5 days *If allergic to penicillin* Clindamycin 300 mg (child: 7.5 mg/kg up to 300 mg) orally, every 8 hours for 5 days	Paracetamol 1000 mg every 4 hours (max 4 g/day) (child: as per TG) *OR* Panadeine Forte (30 mg codeine, 500 mg paracetamol) every 6 hours (max 8 tabs/day) *Can be combined with* Ibuprofen 200–400 mg every 4–6 hours as needed (max 2.4 g/day) (child: 4–10 mg/kg every 6–8 hrs, max 40 mg/kg)

Nonresponsive superficial infections with swelling and/or systemic signs and symptoms	Metronidazole 400 mg (child: 10 mg/kg up to 400 mg) orally, every 12 hours for 5 days and phenoxymethylpenicillin 500 mg (child: 12.5 mg/kg up to 500 mg) orally, every 6 hours for 5 days *OR* Metronidazole 400 mg (child: 10 mg/kg up to 400 mg) orally, every 12 hours for 5 days and amoxycillin 500 mg (child: 12.5 mg/kg up to 500 mg) orally, every 8 hours for 5 days *OR* Amoxycillin + clavulanate 875 + 125 mg (child: 22.5 + 3.2 mg/kg up to 875 + 125 mg) orally, every 12 hours for 5 days *If allergic to penicillin* Clindamycin 300 mg (child: 7.5 mg/kg up to 300 mg) orally, every 8 hours for 5 days	Paracetamol 1000 mg every 4 hours (max 4 g/day) (child: as per TG) *OR* Panadeine Forte (30 mg codeine, 500 mg paracetamol) every 6 hours (max 8 tabs/day) *Can be combined with* Ibuprofen 200–400 mg every 4–6 hours as needed (max 2.4 g/day) (child: 4–10 mg/kg every 6–8 hrs, max 40 mg/kg)

Note: All patients with oral infection should be seen by a dentist within 2 or 3 days. If there is difficulty in breathing, swallowing, or opening the mouth, then urgent referral to ED is necessary and antibiotics will be given IV at the ED.
